# Comparison of multiparametric magnetic resonance imaging sequences with laboratory parameters for prognosticating renal function in chronic kidney disease

**DOI:** 10.1038/s41598-021-01147-z

**Published:** 2021-11-11

**Authors:** Tsutomu Inoue, Eito Kozawa, Masahiro Ishikawa, Daichi Fukaya, Hiroaki Amano, Yusuke Watanabe, Koji Tomori, Naoki Kobayashi, Mamoru Niitsu, Hirokazu Okada

**Affiliations:** 1 Department of Nephrology, Faculty of Medicine, Saitama Medical University, 38 Morohongo, Moroyama-machi, Iruma-gun, Saitama, 350-0451 Japan; 2 Department of Radiology, Faculty of Medicine, Saitama Medical University, 38 Morohongo, Moroyama-machi, Iruma-gun, Saitama, 350-0451 Japan; 3 School of Biomedical Engineering, Faculty of Health and Medical Care, Saitama Medical University, 38 Morohongo, Moroyama-machi, Iruma-gun, Saitama, 350-0451 Japan

**Keywords:** Kidney diseases, Predictive markers

## Abstract

Magnetic resonance imaging (MRI) is playing an increasingly important role in evaluating chronic kidney disease (CKD). It has the potential to be used not only for evaluation of physiological and pathological states, but also for prediction of disease course. Although different MRI sequences have been employed in renal disease, there are few studies that have compared the different sequences. We compared several multiparametric MRI sequences, and compared their results with the estimated glomerular filtration rate. Principal component analysis showed a similarity between T1 values and tissue perfusion (arterial spin labelling), and between fractional anisotropy (diffusion tensor imaging) and apparent diffusion coefficient values (diffusion-weighted imaging). In multiple regression analysis, only T2* values, derived from the blood oxygenation level-dependent (BOLD) MRI sequence, were associated with estimated glomerular filtration rate slope after adjusting for degree of proteinuria, a classic prognostic factor for CKD. In receiver operating characteristic curve analysis, T2* values were a good predictor of rapid deterioration, regardless of the degree of proteinuria. This suggests further study of the use of BOLD-derived T2* values in the workup of CKD, especially to predict the disease course.

## Introduction

There have been many advances in multiparametric magnetic resonance imaging (MRI) in the evaluation of renal physiology and pathology^1^, including studies of the association of quantitative MRI with estimated glomerular filtration rate (eGFR). These MRI sequences are called functional MRI (fMRI) to distinguish them from conventional MRI for evaluating morphology. In longitudinal studies, the close association between T2* (= 1/apparent relaxation rate, R*) values on blood oxygenation level-dependent (BOLD) MRI and GFR changes over time (slope) have led to it being considered an accurate prognostic indicator of chronic kidney disease (CKD) progression^2–4^. In this study, we investigated whether other MRI/fMRI sequences might be useful for estimating the eGFR slope. We used two approaches, first, looking for similarities between MRI sequences using principal component analysis; and second, looking for correlations between MRI measurements and the eGFR slope.

At our institution, patients with CKD usually undergo (1) BOLD fMRI to measure T2* values, (2) arterial spin labelling (ASL) fMRI to measure perfusion volume and T1 signal, (3) diffusion-weighted imaging (DWI) to derive the apparent diffusion coefficient (ADC), (4) diffusion tensor imaging (DTI) fMRI to assess fractional anisotropy (FA) values, and (5) T1-weighted Dixon MRI to assess morphology. We reviewed the scans of CKD patients with declining eGFR who had been under our care for > 1 years to assess correlations between changes in findings in the different MRI sequences and the eGFR slope in CKD.

## Results

### Relationship between eGFR and MRI findings

A total of 151 cases were followed for a median of 3.75 years (Table 1). Multiple regression analysis was performed to evaluate the influence of each explanatory variable on eGFR in patients who had undergone MRI (Table S1). Levels of uric acid and proteinuria were significantly associated with eGFR. Functional MRI parameters were also significantly associated with renal function. On comparison of the standardized partial regression coefficients (SPRCs), the closest correlation with eGFR was seen for the Dixon water gradient, an index of the difference in signal intensity between cortex and medulla in Dixon water images.

**Table 1:** Clinical background of study participants.

Clinical parameter	n	%	Median	Interquartile range
**Total participants**	151			
**Age (year)**			64.0	53.0–72.0
**Gender (female), %**	51	33.8		
**Mean blood pressure (mmHg)**			97.4	92.8–102.1
**Urinary protein creatinine Ratio g/gCr**			0.77	0.19–1.78
**Uric acid (UA) µmol/L**			377.1	331.3–413.4
**Estimated glomerular filtration rate (eGFR) (mL/min/1.73** **m**^**2**^)			42.3	26.8–52.5
**eGFR Slope mL/min/1.73 m**^**2**^** /year**			− 2.04	− 3.90–− 0.72
**Disease aetiology**				
Nephrosclerosis	53	35.1		
Primary glomerular disease	50	33.1		
Diabetic kidney disease	29	19.2		
Tubular interstitial nephritis	4	2.6		
Vasculitis	3	2.1		
Unknown	12	7.9		
**Haemoglobin A1c (HbA1c), % in diabetics**	29		5.51	5.23–6.07

### Relationship among MRI sequences

The cortical-to-medullary gradient does not lend itself to principal component analysis. We looked at quantitative measurements, including the renal cortical signal intensity, to examine the interrelation among different MRI sequences. Although many values correlated significantly with eGFR, they did not necessarily correlate with each other (Table 2). Each MRI sequence evaluates a different aspect of the kidney.

**Table 2:**
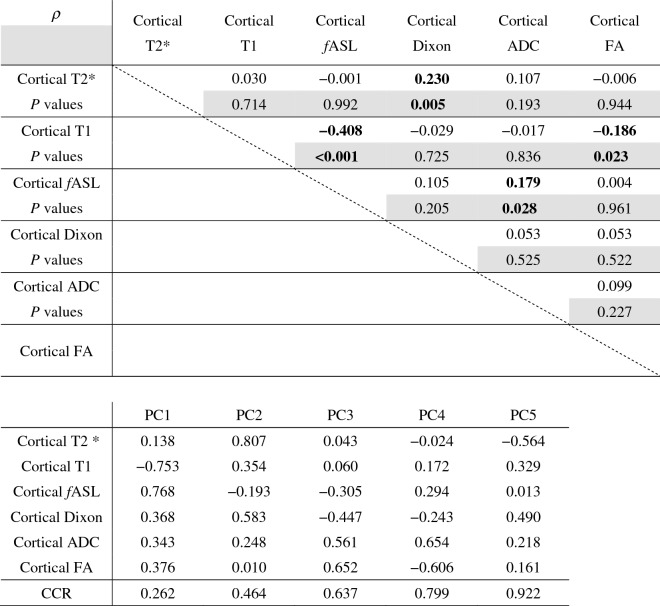
Correlation matrix for MRI values and results of principal component analysis.

*fASL* perfusion volume estimated by arterial spin labelling, *Dixon* signal intensity of Dixon water image, *ADC* apparent diffusion coefficients, *FA* fractional anisotropy, *CCR* Cumulative contribution ratio. P <

0.05 was considered siginficant (bold).

**Figure 1:**
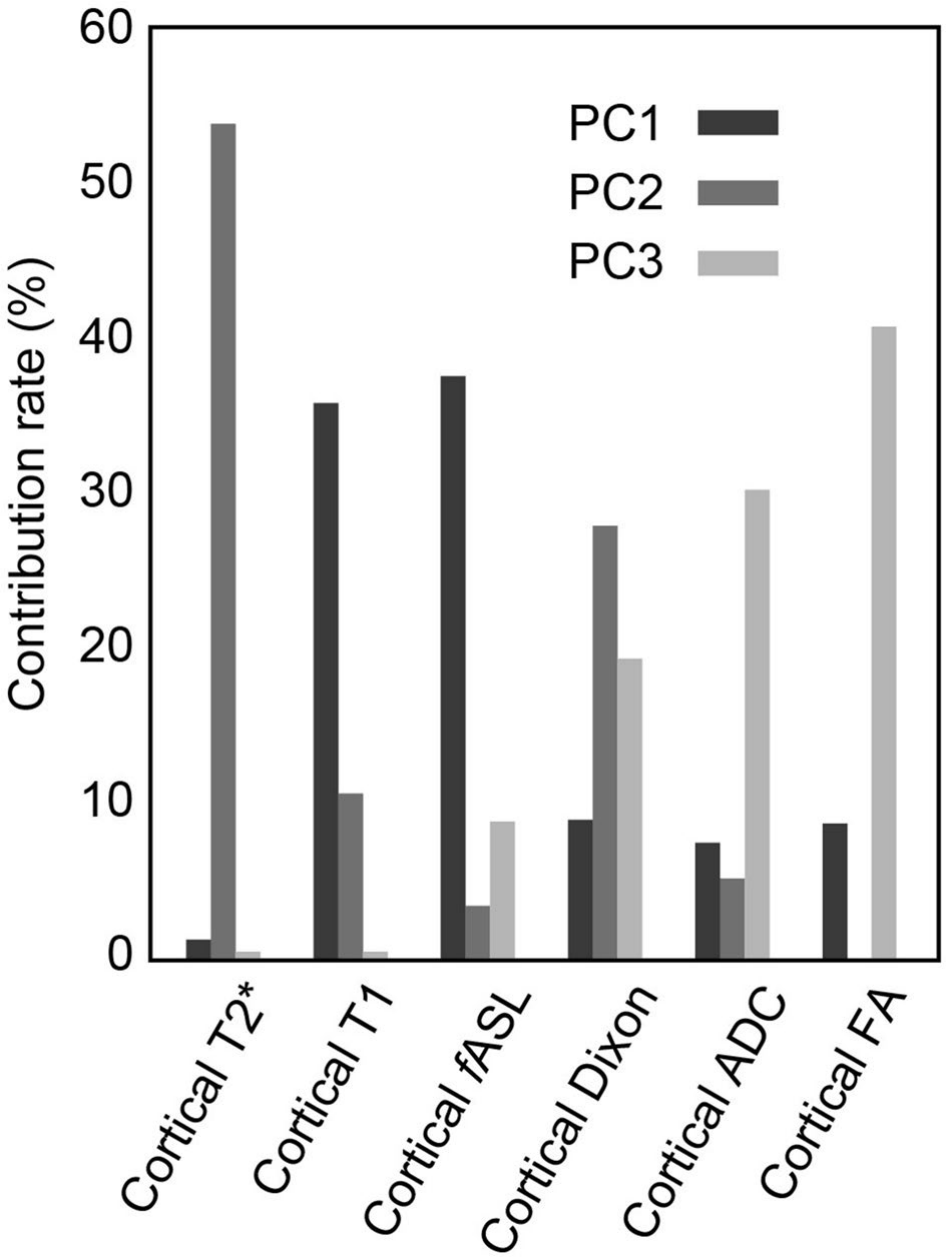
Principal component analysis for each MRI. *PC* Principal component, *fASL* perfusion volume estimated by arterial spin labelling, *Dixon* signal intensity of Dixon water image, *ADC* apparent diffusion coefficients, *FA* fractional anisotropy. PC1 reflects renal blood flow and inflammatory changes. PC2 is an indicator of hypoxia. PC3 is an indicator of fibrosis and associated microstructural degeneration; Dixon water imaging contributed weakly to all three.

Principal component analysis based on the correlation coefficient matrix examining the characteristics of MRI sequences found that principal components (PC) 1–5 had a cumulative contribution of 92%, whereas PC4 and PC5 were similar to PC3 and PC2 (Table 2). We therefore focused on PC1–3. The contribution of each sequence to PC1–3 is shown in Fig. 1.

### Relationship between the eGFR slope and fMRI values

The association between eGFR slope and each variable are shown in Table 3. As expected, proteinuria was a good predictor of eGFR slope. With MRI, cortical T2* values, the T2* gradient, cortical/medullary ADC values, and Dixon water gradient significantly correlated before adjustment for proteinuria in addition to age and gender; we found that cortical T2* values and the T2* gradient were the only explanatory variables independent of proteinuria. In fact, ADC values and the parameters associated with Dixon water images were both significantly associated with proteinuria, suggesting that proteinuria displayed collinearity with them as explanatory variables for eGFR slope (Table S2). Results following adjustment for patient background and laboratory data did not differ from those corrected for proteinuria alone (Table 3). We therefore concluded that cortical T2* values, and the T2* gradient, were the best predictors of rapid decline, independent of proteinuria.

**Table 3:** Multivariate analysis of the association between eGFR slope and individual variables.

	SPRC	*P*	Adjusted by			
			UPCR		UPCR, + mBP, UA, eGFR, DKD	
			SPRC	*P*	SPRC	*P*
mBP	− 0.001	0.986				
UPCR	**− 0.329**	**< 0.001**				
UA	0.039	0.640				
eGFR	0.035	0.677				
DKD	− 0.080	0.332				
Cortical T2*	**0.179**	**0.030**	**0.170**	**0.030**	**0.171**	**0.033**
medullary T2*	0.124	0.136	0.118	0.134	0.099	0.223
T2* gradient	**− 0.266**	**0.001**	**− 0.235**	**0.002**	**− 0.270**	**0.001**
Cortical T1	− 0.119	0.141	− 0.069	0.376	− 0.086	0.278
Medullary T1	− 0.065	0.438	− 0.014	0.865	− 0.032	0.697
T1 gradient	0.107	0.191	0.059	0.451	0.065	0.411
Cortical *f*ASL	0.035	0.664	− 0.065	0.414	− 0.032	0.718
Medullary *f*ASL	− 0.048	0.553	− 0.133	0.090	− 0.121	0.153
*f*ASL gradient	− 0.127	0.120	− 0.032	0.696	− 0.080	0.352
Cortical Dixon	0.115	0.165	0.053	0.511	0.055	0.511
Medullary Dixon	0.094	0.252	0.059	0.450	0.057	0.481
Dixon gradient	**− 0.230**	**0.007**	− 0.093	0.302	− 0.136	0.186
Cortical ADC	**0.180**	**0.024**	0.116	0.134	0.129	0.100
Medullary ADC	**0.179**	**0.025**	0.118	0.127	0.124	0.115
ADC gradient	− 0.048	0.550	− 0.002	0.981	− 0.025	0.757
Cortical FA	− 0.082	0.323	− 0.115	0.144	− 0.107	0.176
Medullary FA	0.032	0.694	− 0.038	0.632	− 0.022	0.794
FA gradient	0.129	0.125	0.072	0.375	0.112	0.210

Multivariate analysis was used for the association between eGFR slope and each variable. *mBP* mean blood pressure, *UPCR* urinary protein creatinine ratio, *UA* uric acid, *DKD* diabetic kidney disease, *fASL* perfusion volume estimated by arterial spin labelling, *Dixon* signal intensity of Dixon water image, *ADC* apparent diffusion coefficients, *FA* fractional anisotropy, *SPRC* standardized partial regression coefficients, P value < 0.05 was considered significant (bold). Objective variable: eGFR slope. All results were adjusted for age and gender.

We next examined whether cortical T2* values and the T2* gradient could provide valid information in addition to that obtained when estimating eGFR slope by multiple linear regression (Table 4). The adjusted R^2^ for the T2* gradient in addition to proteinuria was 0.188. When cortical T2* values were added to them, the adjusted R^2^ was almost identical, at 0.183, although cortical T2* values and the T2* gradient were considered independent of each other in multiple regression analysis.

**Table 4:** Results of multiple regression analysis of the slope of eGFR.

	Explanatory variable					
	UPCR	T2* gradient	T2* cortical	UPCR + T2* gradient	UPCR + T2* cortical	UPCR + T2* gradient + T2* cortical
R^2^	0.158	0.120	0.083	**0.210**	0.185	**0.210**
Adjusted R^2^	0.140	0.102	0.064	**0.188**	0.162	0.183
RMSE	3.373	3.447	3.520	**3.278**	3.330	3.290

The best value is indicated by bold.

Multiple regression analysis was used for the slope of eGFR. *UPCR* urinary protein:creatinine ratio, *RMSE* root mean square error. Objective variable: eGFR slope. All explanatory variables were adjusted for age and gender.

**Figure 2:**
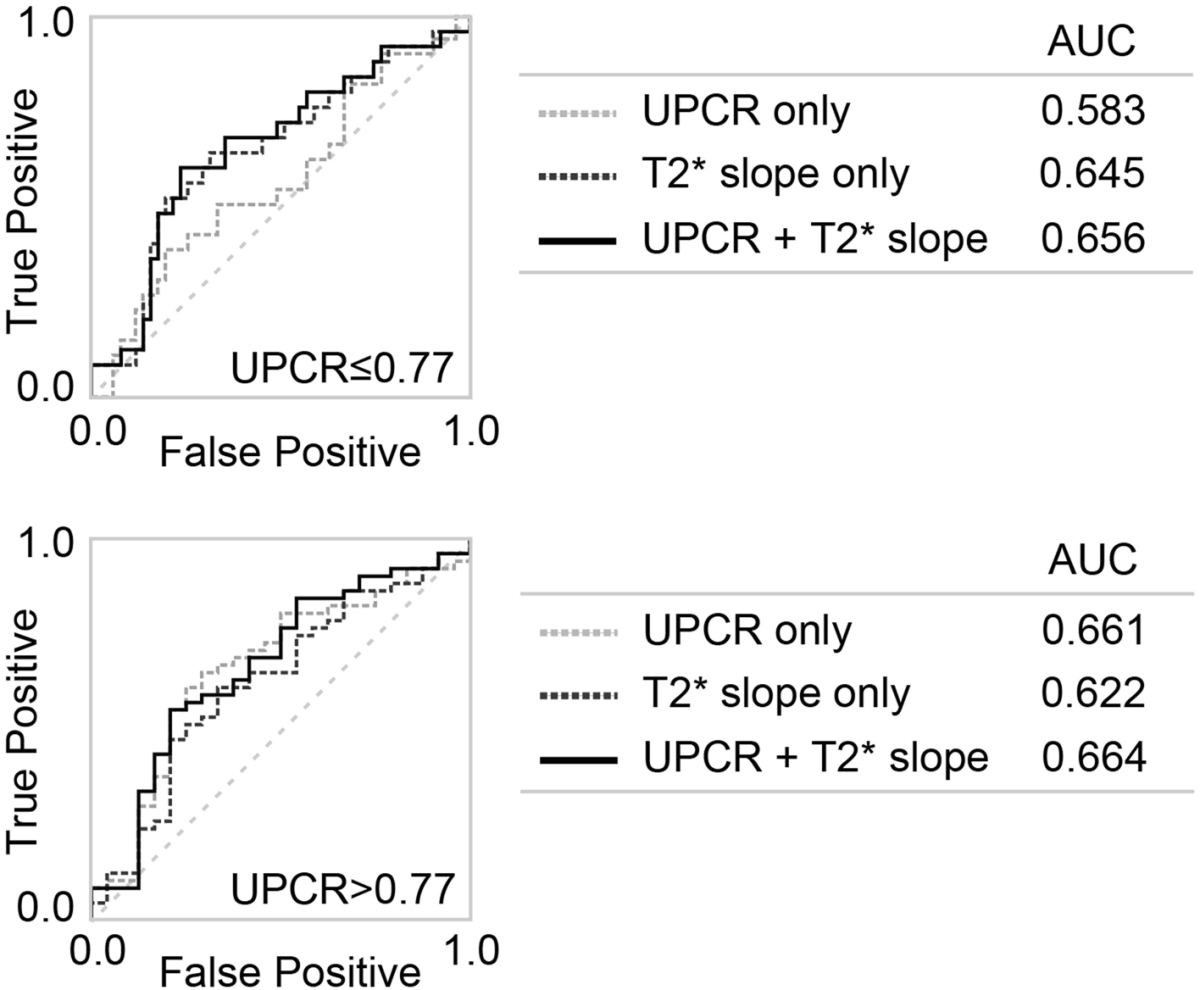
Receiver operating characteristic curve for CKD progression/eGFR decline. Cases were divided into two groups according to degree of proteinuria. *UPCR* urinary protein/creatinine ratio, *AUC* area under the curve.

Next, patients were divided into two groups based on the mean values of proteinuria (cut-off value = 0.77 g/gCr) and actual eGFR slope (cut-off value =  − 2.04). Accuracy in identifying the rapidly progressing group was examined by receiver operator characteristics (ROC) curves (Fig. 2). In the low-proteinuria group, area under the curve (AUC) of proteinuria alone was not sufficient; the T2* gradient tended to be more accurate. In contrast, in the group with high proteinuria, either proteinuria alone or T2* gradient alone was sufficiently accurate; the additive contribution of T2* gradient to proteinuria was not significant. As a single indicator, T2* gradient appeared a better indicator of rapid eGFR decline and progression.

**Figure 3:**
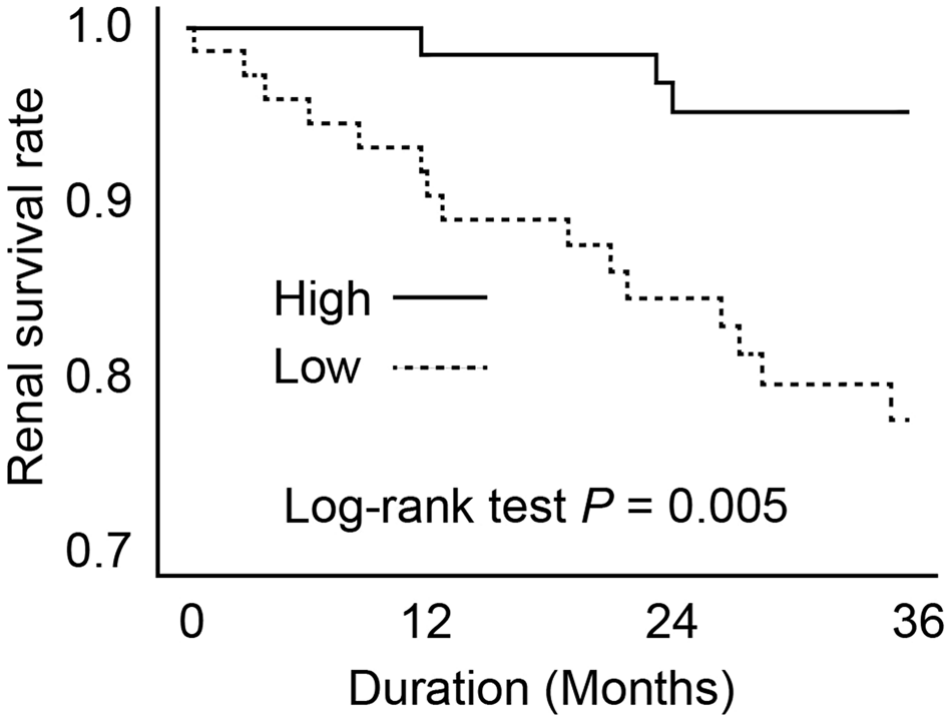
Kaplan–Meier curve with the introduction of renal replacement therapy due to renal death as the endpoint. ‘High’ and ‘Low’ correspond to the magnitude of the gradient of T2* value of the inner and outer layers of the kidney.

Patients were then divided into two groups based on mean values of the T2* gradient (cut-off value =  − 8.23 × 10^–2^) and actual renal prognosis was compared between the groups, T2* gradient ‘High’ and ‘Low’. In this study, ‘High’ implies a large difference in T2* signal between cortex and medulla, while ‘Low’ implies a small difference. Three years after first undergoing MRI, 18 cases had started renal replacement therapy (RRT). Median time to initiation of RRT was 16.3 months. The Kaplan–Meier curve and 2-sided log-rank tests are shown in Fig. 3. RRT was started in 15 patients in the T2* gradient ‘Low’ group but only 3 patients in the ‘High’ group, with this difference being statistically significant in the log-rank test (*P* = 0.005).

## Discussion

Few longitudinal studies have comprehensively evaluated multiparametric MRI sequences including BOLD MRI, DWI, and others to evaluate CKD^5,6^. We found that BOLD MRI has peculiar properties, and may be evaluating physiological aspects of the kidney that are different from those evaluated by other MRI sequences. Only BOLD MRI T2* values showed a significant correlation with the eGFR slope, and, thus, the course of the disease, independent of proteinuria.

We previously reported the clinical meaning of T2* and ADC values^3,7^. In this study, we characterised each MRI sequence to determine which sequence(s) would be useful for assessing the prognosis of renal disease.

We first examined the relationship between each quantitative MRI measurement and eGFR, an indicator of renal function, at the time of imaging. We examined the similarities of each MRI sequence using principal component analysis based on the correlation coefficient. We found that each sequence captures different aspects of renal pathology. Defining ‘annual rate of decline in renal function = eGFR slope’ as an index of prognosis of renal disease, our aim was next to see if there were any other useful MRI sequences predictive of eGFR slope. Since eGFR slope is a direct marker of prognosis of renal disease, but not a hard endpoint in clinical research, we re-evaluated our findings using end-stage renal disease requiring institution of renal replacement therapy as a hard endpoint.

As shown in this study, in particular, a T2* map generated with BOLD MRI has distinctly different properties from those of other sequences, because T2* values are not strongly associated with any laboratory parameter or clinical finding. BOLD MRI seems to be the only sequence that can be used to predict the prognosis of CKD, as already reported^2–4^. Renal fibrosis and tubulointerstitial alteration have long been thought to be associated with the prognosis of renal disease. Because ADC and FA values would be expected to quantify these pathological changes, PC3, where ADC and FA values are the main factors, should have a meaningful relationship with the eGFR slope; however, there was no significant correlation, similar to our previous report^3^.

Findings on multiparametric MRI sequences significantly correlated with eGFR. Interestingly, when focusing on the relationship among the values, the behaviour of both ADC and FA values obtained from images based on the diffusion phenomenon of water molecules was similar. Furthermore, the non-fMRI, T1-weighted Dixon images showed findings different from those of fMRI, as expected from the imaging principle. When renal function declines, atrophy of the cortical layer progresses and is accompanied by concomitant changes in multiparametric MRI. We and others have observed that all MRI sequences capturing such morphological and physiological degeneration of the kidney reveal decreased difference between the values in the cortex and those in the medulla (as is often seen in ultrasound), resulting in decrease of the cortical-to-medullary ‘gradient’. On the other hand, it is important to note that the signal intensity of each region, especially for T1-weighted Dixon images, cannot be regarded as a quantitative value reproducible on multiple scanners of different manufacture.

Attention has now focused on multiparametric MRI; we expect that our results will be useful as basic data in combining imaging methods and evaluating the kidney from multiple perspectives, with consideration to the characteristics of each MRI sequence.

This study has a number of limitations. Patient conditions at the time of MRI imaging were heterogeneous, as diuretic doses were not uniform, and patients still relied on oral hydration. Diuretics are known to change T2* values in the renal medulla. Also, the tissue contrast in other MRI examinations without exacting rules for fasting and water consumption could be affected by patients’ differing hydration levels.

Before MRI imaging, however, the patients skipped one meal and were required to drink water, satisfying a prescan requirement in the consensus protocol^8^. Further, the effects of diuretics on T2* gradient, T2* values in the cortex^2^ and on other MRI values are reportedly limited. T2* values may differ depending on the underlying disease, even for the same eGFR^7^. Therefore, unlike other MRI sequences that show a uniform correlation with eGFR, the utility of T2* may depend on subject characteristics^9^. In this study, eGFR estimated from creatinine was used as an index of renal function. The correlation coefficient between eGFR and GFR, which is measured as inulin clearance, is approximately 0.8^10^. Nuclear medicine techniques with Cr-51 ethylenediaminetetraacetic acid (Cr-51-EDTA) or Tc-99 m diethylenetriamine pentaacetic acid (Tc-99 m-DTPA) may be more accurate.

Dynamic contrast-enhanced magnetic resonance imaging (DCE-MRI) theoretically could have been used in this study solely for the purpose of assessing GFR at the time of imaging^11,12^. We found that ASL, which also assesses tissue perfusion, actually had a higher correlation with eGFR (Table S1), without the need for gadolinium contrast and its attendant risks in CKD patients. We did not include DCE-MRI in this study because we wanted to examine imaging methods that could be repeated as needed in patients with impaired renal function.

## Conclusion

In this study, we examined multiparametric MRI sequences and their ability to predict the clinical course of CKD. T2* map generated by BOLD MRI was found to be the most accurate for estimating eGFR slope and predicting CKD prognosis independent of proteinuria levels at the time of scanning.

## Methods

### Ethical approval and consent to participate

The study was conducted at a single centre under a retrospective observational design without subject invasion or intervention. The study used only existing information, such as clinical data including MRI, and was based on the guidelines for research in Japan. Under these guidelines, information on the full scope of the study, such as subjects, purpose, and survey content, are required to be made public and the opportunity to refuse participation must be granted in place of obtaining written informed consent. The Institutional Review Board (IRB) of Saitama Medical University approved this research and its publication in accordance with the Declaration of Helsinki and the Japanese guidelines for clinical research (Approval No. 19048.01 01/07/2019). The protocol ID is R000042466 in UMIN-CTR (University Hospital Medical Information Network-Clinical Trials Registry; https://www.umin.ac.jp/ctr/index.htm), which has been officially recognized as a registered site that meets ICMJE (International Committee of Medical Journal Editors) standards.

### Participant characteristics

CKD was diagnosed on the basis of persistent proteinuria or chronic decline in estimated glomerular filtration rate (eGFR) (< 60 mL/min/1.73 m^2^). From a total of 249 CKD patients who underwent magnetic resonance imaging (MRI) between April 2013 and June 2019, those cases with a sufficient number of visits for the treatment and evaluation of CKD (more than three times during a period of at least 1 years) were enrolled (n = 186). We excluded subjects^7^ who had already received renal replacement therapy such as dialysis or transplantation (n = 4); those under the age of 18 years (n = 3); those diagnosed with primary nephrotic syndrome (n = 13), acute kidney injury (n = 5), or hydronephrosis (n = 1); and those without urinary protein values or blood tests (n = 9), leaving a total of 151 cases. The median and interquartile range of the observation period was 3.75 (2.14, 6.08) years.

We obtained clinical information from electronic health records and evaluated the following data: mean blood pressure (mmHg) = diastolic pressure + (systolic pressure – diastolic pressure)/3; eGFR calculated by the formula for Japanese^10^, (eGFR (mL/min/1.73 m^2^) = 194 × Cr^−1.094^ × age^−0.287^ (× 0.739 for female cases). Urinary protein levels were noted and corrected for urinary creatinine (urine protein:creatinine ratio, g:g). The time when the MRI was performed was included as part of the observation period. During the observation period data were sampled every 3–6 months, and their mean values were used as an explanatory variable. We estimated the rate of decline in eGFR using a linear approximation method applied to serum creatinine.

### MR examination

MR examinations were performed on a 3.0 T (Skyra, Siemens Healthcare, Erlangen, Germany) scanner using a spine coil and 18-channel phased-array body coil. Images were acquired in an oblique coronal plane for BOLD, ASL, and the map of T1 relaxation times; and in the coronal plane DTI and Dixon imaging. The left kidney was chosen for quantitative evaluation of oblique-coronal images because artefacts due to respiratory motion and colon gas were less pronounced on the left side. Images were analysed on an MR system (Syngo software, Siemens Healthcare, Erlangen, Germany) for BOLD, DTI, Dixon imaging, and T1 map and on an external workstation using MATLAB (version 2020a, Mathworks, Natick, MA, USA) for ASL.

For BOLD MRI, 12 T2*-weighted images corresponding to the 12 different gradient echoes were acquired. T2* maps were generated on a pixel-by-pixel basis by fitting a linear regression method through the logarithms of the signal intensities versus their 12 echo times^7^. For T1 mapping, a T1-weighted volume interpolated breath hold examination (VIBE) sequence with flip angles 4° and 21° was employed. To correct B1 heterogeneity, a B1 mapping pulse sequence was used to generate a B1 field map of the entire abdomen using the stimulated echo/spin echo method^13^. ASL MRI was performed using a flow-sensitive alternating inversion recovery (FAIR) perfusion preparation with a steady state free precession (True-FISP) pulse sequence^14^. Perfusion was determined on a pixel-by-pixel basis with the following formula^15^:

$$f=\frac{\lambda }{2TI}\frac{\Delta M(TI)}{{M}_{0}}exp\frac{TI}{T1}$$

where *f* is renal blood flow, λ is the constant tissue-blood partition coefficient (0.8 mL/g), ΔM is the result of subtraction of the selective and non-selective inversion images, TI is 1200 ms, and M_0_ is the equilibrium magnetization. The renal cortical T1 values of each patient were entered into the formula. For DTI, a fat-saturated echo-planar sequence was applied with the following imaging parameters: 6 diffusion directions, and b values of 0, 300, and 600 s/mm^2^ with respiratory gating. The diffusion measurements along 6 axes were fitted to a 3 × 3 symmetric matrix, which was defined as the diffusion tensor. The amount of diffusion anisotropy was calculated and a parameter map of fractional anisotropy (FA-map) was generated, scaled from 0 (no preferred diffusion direction, isotropic diffusion) to 1 (only one diffusion direction; completely anisotropic diffusion) according to the methodology of Hueper et al.^16^. ADC maps were generated based on a monoexponential fitting model. Coronal 3D T1-weighted VIBE Dixon images were generated from in-phase, out-of-phase, fat-only, and water-only image data^17^. The signal intensity of water-only images was used because it rendered the clearest images of the kidneys. Total acquisition time was approximately 30 min. The MRI scan parameters are summarized in Table 5.

**Table 5:** MRI scanning parameters.

	BOLD	T1 map	FAIR-ASL	T1W DIXON	DTI
Sequence	2D GE	2D GE	2D GE	3D GE	2D SE
Fast imaging	EPI	EPI	EPI	–	EPI
Echoes	12	1	1	2	1
TE1; DTE (ms)	4.92; D2.46	26	1.32	2.46; D1.23	70
TR (ms)	175	15	2000	5.35	1100
FA (°)	50	4, 21	70	10	–
Half scan	–	–	–	–	0.8
Orientation	Oblique	Oblique	Oblique	Coronal	Coronal
Slices	5	7	1	48	15
Voxel size (mm)	1.4 × 1.4 × 5.0	1.3 × 1.3 × 3.0	1.1 × 1.1 × 5.0	1.1 × 1.1 × 3.0	1.4 × 1.4 × 3.0
FOV (mm)	360 × 360 × 27	360 × 360 × 144	244 × 244 × 34	360 × 360 × 144	360 × 360 × 45
Recon matrix	256	256	256	320	128
Parallel imaging; SENSE factor	3	3	1.5	3	2
Respiratory compensation	Breath hold	Breath hold	Synchronized breathing	Breath hold	Free breathing
Remarks		3D FLASH	TI = 1300	DIXON recon of water, in, and out	b-values: 0, 200, 400, 600

*BOLD* blood oxygenation level dependent, *FAIR-ASL* flow attenuated inversion recovery arterial spin labelling, *T1W* T1 weighted, *DTI* diffusion tensor imaging, *GE* spoiled gradient echo, *SE* spin-echo, *EPI* echo planar imaging, *TE* echo time, *TR* repetition time, *FA* flip angle, *FOV* field of view, *TI* inversion time, *recon* reconstruction.

### TLCO method

This method has been previously described^2^ and its reproducibility is documented^5^. In brief, the outer and inner boundaries of the renal parenchyma were manually drawn on one of the T2* weighted images to serve as an anatomical region of interest template (T1-weighted Dixon image), using custom MATLAB code. The algorithm output 12 T2* values representing the 12 layers. We used these 12 measurements to estimate the corticomedullary T2* value gradient and whole-kidney values based on the following definitions: cortical (mean of the first 3 superficial layers), medullary (mean of layers 8–10), and the resulting gradient (linear fit to the data points 4–7 when plotting T2* vs. % of depth). Images were independently analysed by two observers, who were blinded to the laboratory data to eliminate bias. To assess repeatability, inter-observer coefficients of variation (CoV) were calculated as follows:

$$CoV=100\%\times \sqrt{\frac{1}{2N}\sum_{n=1}^{N}\frac{{({x}_{1,n}-{x}_{2,n})}^{2}}{{\overline{x} }_{n}^{2}}}$$

*CoV* was 4.44%, and the intraclass correlation coefficient, ICC (2, 1), was 0.822 (95% confidence interval 0.689–0.896) for the mean of T2* values in the 12 layers corresponding to the entire kidney.

### Statistical analysis

All values are expressed as median and IQR (interquartile range). We performed multiple linear regression analysis (Tables 2, 4, S1, and S2) with the goal of identifying independent predictors of the annual rate of change in eGFR (eGFR slope) and eGFR. *P* values < 0.05 were considered statistically significant. All statistical analyses, using commercial software (JMP version 15.0, SAS Institute, Cary, NC, USA), Excel (Microsoft, Redmond, WA, USA) including multiple linear regression analysis, multivariate correlation, principal component analysis, multiple regression analysis, and ROC curves, analysed all variables except ICC. SPSS version 26 (IBM, Armonk, NY, USA) was used to evaluate ICC.

## Supplementary Information

Supplementary Information.
