# Supplementary material for: Comparison of multiparametric magnetic resonance imaging sequences with laboratory parameters for prognosticating renal function in chronic kidney disease
Source: Sci Rep. 2021 Nov 11;11:22129. doi: 10.1038/s41598-021-01147-z (PMC8586015; doi:10.1038/s41598-021-01147-z)
Supplement: Supplementary file 1 — Supplementary Information. [file 41598_2021_1147_MOESM1_ESM.docx]

**Supplementary Tables**

|  | SPRC | *P* |
| --- | --- | --- |
| mBP | −0.103 | 0.197 |
| UPCR | **−0.326** | **< 0.001*** |
| uric acid | **−0.268** | **0.001*** |
| DKD | 0.060 | 0.462 |
| Cortical T2* | 0.120 | 0.141 |
| Medullary T2* | 0.024 | 0.774 |
| T2* gradient | **−0.243** | **0.002*** |
| Cortical T1 | **−0.239** | **0.002*** |
| MedullaryT1 | **−0.258** | **0.001*** |
| T1 gradient | 0.114 | 0.159 |
| Cortical *f*ASL | **0.450** | **< 0.001*** |
| Medullary *f*ASL | **0.405** | **< 0.001*** |
| *f*ASL gradient | **−0.380** | **< 0.001*** |
| Cortical Dixon | **0.270** | **0.001*** |
| Medullary Dixon | 0.147 | 0.073 |
| Dixon gradient | **−0.547** | **< 0.001*** |
| Cortical ADC | **0.176** | **0.025*** |
| Medullary ADC | 0.119 | 0.134 |
| ADC gradient | **−0.226** | **0.004*** |
| Cortical FA | 0.130 | 0.112 |
| Medullary FA | **0.333** | **< 0.001*** |
| FA gradient | **0.429** | **< 0.001*** |

**Table S1** Results of multiple linear regression analysis, objective variable is eGFR at the time of MRI scan.

SPRC: standardized partial regression coefficients, mBP: mean blood pressure, UPCR: urinary protein:creatinine ratio, DKD: diabetic kidney disease, *f*ASL: perfusion volume estimated by arterial spin labeling, Dixon: signal intensity of Dixon water image, ADC: apparent diffusion coefficients, FA: fractional anisotropy. Objective variable: eGFR at time of MRI scan. All results were adjusted for age and gender.

|  | SPRC | *P* |
| --- | --- | --- |
| Cortical Dixon | **−0.191** | **0.021*** |
| Medullary Dixon | −0.106 | 0.199 |
| Dixon gradient | **0.464** | **< 0.001*** |
| Cortical ADC | **−0.209** | **0.009*** |
| Medullary ADC | **−0.198** | **0.014*** |
| ADC gradient | 0.142 | 0.083 |

**Table S2** Results of multiple linear regression analyses.

The objective variable is the urinary protein:creatinine ratio (UPCR); explanatory variables are the ADC values and indices for Dixon water images. All results were adjusted for age and gender. SPRC: standardized partial regression coefficients, ADC: apparent diffusion coefficients
